# Exploring the dynamics of a single vesicle induced by Fe₃O₄ nanoparticles using micropipette manipulation

**DOI:** 10.1371/journal.pone.0327639

**Published:** 2025-07-07

**Authors:** Nazia Ahmed, Tawfika Nasrin, Mohammad Abu Sayem Karal

**Affiliations:** 1 Department of Physics, Bangladesh University of Engineering and Technology, Dhaka, Bangladesh; 2 Department of Mathematical and Physical Sciences, East West University, Dhaka, Bangladesh; Brandeis University, UNITED STATES OF AMERICA

## Abstract

Magnetite nanoparticles (MNPs, Fe_3_O_4_) have gained substantial interest for different biomedical and biochemical applications. Therefore, it is important to understand the mechanism of interaction between MNPs and cell membranes. As a model for cells, giant unilamellar vesicles (GUVs) are used in various research studies, providing valuable insights into the behavior of lipid bilayers and their interactions with MNPs. To understand the mechanism of interaction between MNPs and membranes, the dynamics of a ‘single GUV’ are explored using the micropipette technique under physiological conditions. The GUVs exhibited deformation upon adsorption of anionic MNPs into the membrane, with the degree of deformation (e.g., compactness) increasing over time. The addition of MNPs through a micropipette into the vicinity of a ‘single GUV’ induced various shape changes; for example, a prolate shape transformed into two spheres connected by a neck. The fraction of the shape changes of GUVs increased with the concentration of MNPs. These results indicated that MNPs were absorbed onto the outer monolayer, inducing an area mismatch between the outer and inner monolayers of the membrane. The change in membrane area upon MNP binding in a ‘single GUV’ was investigated using the micropipette aspiration technique. Initially, the membrane area increased at a faster rate until reaching a saturation point, then decreased at a slower rate back to the original point, followed by a slight increase at a very slow rate. These changes suggest rapid stretching, slower compression, and finally slow stretching in the membranes of the GUV. Based on these results, we discuss the interaction mechanism of anionic MNPs with a single GUV.

## 1. Introduction

Nanoparticles (NPs) have an extensive range of applications, including drug delivery, cosmetics, antibiotics, and bioimaging [1]. Given that some products containing NPs are intended for direct human use—such as food, cosmetics, and drug delivery—they are likely to come into contact with biological systems. Furthermore, these NPs will inevitably be released into the environment at various stages of their life cycle, including manufacturing, usage, and disposal. On the other hand, NPs enter our bodies through inhalation, intraperitoneal, intravenous, subcutaneous, dermal and oral routes, and damage the healthy cells by interacting with their membranes [2]. Hence, there is an increasing interest in exploring the toxic potential and environmental effects of NPs [3,4]. Among the various types of NPs, magnetite nanoparticles (MNPs, Fe_3_O_4_) are found in the environment due to both natural processes such as volcanic eruptions and meteorite impacts and human activities such as industrial activities, power plants, and marine sediments deposition [5,6].

Studies have indicated that their emissions may pose risks to human health and the environment [7]. The properties and toxicity of these particles can vary based on factors like size, shape, and surface coating [8]. Notably, MNPs can be easily prepared by green synthesis technique [9]. The study found the abundance of this particle in the human brain, which is critically linked to neurological diseases such as Alzheimer’s [10]. It has also demonstrated that MNPs can interact with various blood components, potentially leading to adverse effects such as hemolysis, thrombosis, and platelet aggregation depending on their size, surface charge, and coating [11]. Studies have shown that MNPs can cause hemolysis, particularly due to their interaction with cell membranes and the generation of reactive oxygen species. Factors like surface charge, size, and coating significantly affect the extent of hemolysis [12].

To study the interaction of NPs as well as different types of toxins and peptides with cell membranes, instead of real cells, artificially synthesized giant unilamellar vesicles (GUVs), almost similar in size to real cells, are very popular in the scientific arena [13–15]. On the other hand, as the structure of cell membranes is complex, researchers have focused on studying the GUVs as an alternative to real cells [16]. GUVs are emerging as a promising model for studying the interaction of NPs with cell membranes, as they can be easily synthesized and tailored to meet specific experimental needs. Numerous studies have explored the interactions between NPs and lipid membranes. One study examined the effects of AuNPs on lipid packing and pore formation, revealing that AuNPs increased membrane fluidity while reducing the circularity of lipid vesicles [17]. It was also examined the changes in shape of GUVs induced by charged core shell magnetic NPs [18]. Another study examined the uptake of silica NPs of varying sizes by DMPC lipids and found that the NPs could penetrate cells and localize within the membrane, resulting in alterations to membrane structure and protein distribution [19]. The study of these adverse effects at physiological conditions has become a key area of interest in biophysics [20,21].

So far, a series of studies on the interaction of anionic Fe_3_O_4_ with GUVs have been done by the author’s group by varying lipid composition and buffer salt concentration [22], buffer sugar concentration [23], membrane cholesterol [24], polymer-grafted lipid [25], and membrane potential [26]. A recent review discussed how various elements change the vesicle deformation and lipid membrane poration induced by anionic MNPs [27]. The head of DOPC lipid is a dipole of P^−^−N^+^, hence anionic MNPs bind with N^+^ [22,28]. The presence of a small amount (e.g., 0.01% of the total lipids) of channel forming protein in the membranes of DOPG/DOPC-GUVs changes the membrane permeation [26] along with the electroporation kinetics [29]. However, research on the single-vesicle dynamics induced by NPs is still in its early stages [30]. To reveal the mechanism of interaction between particles (e.g., protein/peptide/neurosteroid) with lipid membranes, it is important to investigate the membrane area change upon binding them using the micropipette aspiration technique [31–33]. This technique is often used in conjunction with optical microscopy, allowing researchers to observe and manipulate a single cell/vesicle or other small structures in real time [34,35]. With this technique, individual cells/vesicles can be manipulated without damage, making it observe the single-vesicle dynamics. The shape changes of GUVs due to the insertion of lipopolysaccharide were investigated using this technique [36–38]. Moreover, it was used to study the effects of neurosteroids on lipid bilayers by applying controlled negative pressure [33].

In this research, we first investigated the deformation of a ‘single GUV’ by the adsorption of MNPs. Then the changes in shape and the reversibility of GUVs have been investigated. Finally, the method of micropipette aspiration [34] has been utilized to monitor the membrane area change of the GUVs. We explore the mechanisms of the dynamics of deformation and shape changes of a ‘single GUV’ upon the adsorption and desorption of MNPs in the membranes. This research could deepen the understanding of the development of safer NPs and advance the field of nanomedicine.

## 2. Materials and methods

### 2.1. Chemicals and reagents

1,2-dioleoyl-*sn*-glycero-3-phosphocholine (DOPC), 1,2-dioleoyl-*sn*-glycero-3 phosphoethanolamine-*N*-[poly-(ethylene glycol)] (PEG2K-DOPE), and 1, 2-dioleoyl-*sn* glycero-3-phospho-(1′-*rac*-glycerol) (sodium salt) (DOPG) were purchased from Avanti Polar Lipids Inc. (Alabaster, AL). Bovine serum albumin (BSA), 1,4-Piperazinediethanesulfonic acid (PIPES), Ethylene glycol-bis(2-aminoethylether)-*N*,*N*,*N*′,*N*′-tetraacetic acid (EGTA), and all other chemicals, such as sodium hydroxide (NaOH), sodium chloride (NaCl), glucose, and sucrose were bought from Sigma-Aldrich (Germany). Ferric chloride anhydrous (FeCl_3_) and ferrous chloride tetrahydrate (FeCl_2_.4H_2_O) were bought from Merck (Germany).

### 2.2. Synthesis of MNPs

The green synthesis method was used to synthesize the *Ipomoea Aquatica* leaf extracts mediated MNPs [9]. *Ipomoea aquatica* leaves, available in Bangladesh, were purchased from the local market (BUET Market, Polashi, Dhaka, Bangladesh). It was not necessary to obtain permission from any authority to purchase these leaves. The prepared sample exhibited a cubic inverse spinel structure with a particle size of approximately 18 nm [9]. The zeta potential of the MNPs was measured at −21.3 mV, indicating their anionic nature [22]. *Ipomoea aquatica* leaf extracts were used as reducing and capping agents for the MNPs. A detailed description of the leaf extracts preparation, the synthesis procedure for MNPs, particle size determination using dynamic light scattering (DLS), and zeta potential measurements can be found in the Supporting Information (S1 in S1 File). Various concentrations (e.g., 0.1, 0.5, 1.0, 3.0, 4.4, 4.5 µg/mL) of MNPs were prepared for different experiments. Since the synthesis conditions were identical for all concentrations, the resulting MNPs maintained the same size within the experimental error (see S2 in S1 File).

### 2.3. Formation and observation of GUVs

GUVs of lipid membranes such as DOPC/PEG2K-DOPE (molar ratio, 99:1) and DOPG/DOPC (molar ratio, 40:60) were prepared in a physiological buffer (10 mM PIPES, 150 mM NaCl, pH 7.0, 1 mM EGTA) by the natural swelling method. In this method, at first, we put 200 μL of a mixture of 1 mM DOPC/PEG2K-DOPE or 1 mM DOPG/DOPC into a 4.5 mL glass vial and dried it under a stream of N_2_ gas to produce a thin, homogeneous lipid film. The solvent was completely removed by keeping the vial in a vacuum desiccator for more than 12 h. Next, 20 μL MilliQ water was added into the glass vial and the mixture was incubated at 45 °C for 8 min (pre-hydration). The hydrated lipid film was then incubated with 1 mL of the buffer containing 0.10 M sucrose for 2–3 h at 37 °C. During incubation, various types of GUVs were formed. The fractions of spherical, prolate (non-axisymmetric), prolate (axisymmetric), fetal (curl), and cylindrical vesicles were approximately 50%, 15%, 15%, 10%, and 10%, respectively, in DOPC/PEG-DOPE (99/1)-GUVs. For getting spherical shaped GUVs, the suspension of the vesicles was centrifuged at 13000 × *g* (*g* is the acceleration due to gravity) at 20 °C for 20 min to separate the lipid aggregates from the suspension of vesicles. These purified GUV suspensions were taken into a handmade microchamber, which was coated with 0.10% (w/v) BSA suspended in buffer containing 0.10 M glucose. Thus, the concentration of internal solution (0.10 M sucrose) and external solution (0.10 M glucose) remained constant to avoid the osmotic effect of vesicles. We observed GUVs in an inverted phase contrast and differential interference contrast (DIC) microscope (IX-73, Olympus, Japan) with a 20 × objective at 25 ± 1 °C controlled by a thermo-controlled stage system (TPi-110R13, TOKAT). The images were recorded using a charged-coupled device (CCD) camera (Olympus DP22, Japan) with a video recorder of 25 fps.

### 2.4. Deformation and compactness of a GUV

At first, a 160 μL suspension of purified GUVs was taken into a handmade U-shaped silicone-rubber microchamber and waited for 20 min for the GUVs to settle down at the bottom of the microchamber. The microchamber is positioned on the thermo-controlled stage of the microscope, as shown in Supporting Information (S3 in S1 File). Then 80 μL MNPs of concentration 13.2 µg/mL were inserted slowly using a pipette into the suspension, resulting in a final MNPs concentration of 4.4 µg/mL in the microchamber. Immediately after inserting the MNPs, we focused on a ‘single GUV’ and observed the time course of the change of GUV using an inverted phase contrast microscope. The targeted GUV was kept in focus during the whole observation period. The dynamics of the GUV were recorded using the CCD camera and later the images at different time points were prepared from the recorded video. Due to the insertion of MNPs, the GUV became deformed, and the degree of deformation changed over time. The compactness (*C*_*om*_) is calculated to measure the deviation of the GUV from its circular shape. Mathematically, *C*_*om*_ is expressed as follows [22,39]:


$$C_{om}=\;\frac{P^{2}}{4\pi S_{\text{cr}}}$$ (1)

where *P* is the perimeter and $S_{cr}$ is the cross-section area of a GUV. For the perfectly spherical shaped GUV, *C*_*om*_ is 1.0. The increase in this value indicates the deviation from its spherical shape. An image processing tool MATLAB was used to analyze the data from recorded image.

### 2.5. Shape change of a GUV induced by spontaneous insertion of MNPs

**Fig 1:**
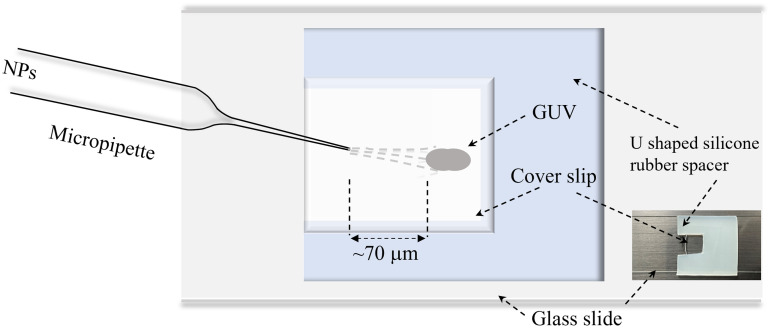
Inducing the solution of MNPs in the vicinity of a GUV using a micropipette.

To observe the change of shape of a ‘vesicle, we followed the ‘single GUV’ method [16]. The same method was used for various types of experiments [13,40,41]. In this experiment, we used an unpurified suspension of GUVs. First, 280 μL of buffer containing 0.10 M glucose was added to the microchamber (Fig 1), followed by the insertion of 20 μL of the unpurified vesicle suspension. It was waited for 20 min to allow the GUVs to settle at the bottom of the microchamber. We focused on a prolate or fetal or cylindrical shaped GUV, and then the solution containing 1.0 μg/mL MNPs was continuously provided to the vicinity of the GUV through a micropipette with tip diameter of ∼20 μm. The distance between the GUV and the tip of the micropipette was ∼70 μm, and Δ*P* = *P*_out_ − *P*_in_ = −30 Pa, where *P*_out_ and *P*_in_ are the pressure of the outside and the inside of a micropipette, respectively, as shown in (Fig 1).

The position of the tip of the micropipette was controlled by a hydraulic micromanipulator (MMO-203, Narishige, Tokyo, Japan) [37]. A glass micropipette was prepared as follows: at first, a glass tube with a 1.0 mm diameter (G-1, Narishige, Japan) was pulled using a puller (PC-100, Narishige Micropipette Puller, Japan) and then cut it by quick fracture at the desired tip diameter. Then, the micropipette aspiration method was used to fill the micropipette with various concentrations of MNPs by using a suction pump. The process of injection pressure of MNPs was controlled by adjusting the height of the vertical column of water in the U-shaped glass tube to which the micropipette was hydraulically connected [37]. We observed the interaction of a ‘single GUV’ with 0.1, 0.5, 1.0, 3.0, and 4.5 μg/mL MNPs for 8 min after starting to add the MNPs solution from the micropipette. Since the micropipette tip is very close to the GUVs and the MNPs diffuse rapidly around the GUVs via a diffusion process, it was assumed that the MNPs concentration around the GUVs is the same as that of the micropipette.

After applying the MNPs, we investigated whether the ‘single GUV’ changes the shape or not. If the GUV changes shape, the reversibility of the shape change is also observed. Next, we focused on a second GUV in the same microchamber and performed similar experiments. The process was repeated for several individual GUVs. Next, the fraction of shape change (*Fr*_s_) was calculated, which is the ratio of shape changed GUVs to the total observed GUVs within the time frame of observation.

### 2.6. Measurement of fractional change in area of a GUV induced by MNPs

**Fig 2:**
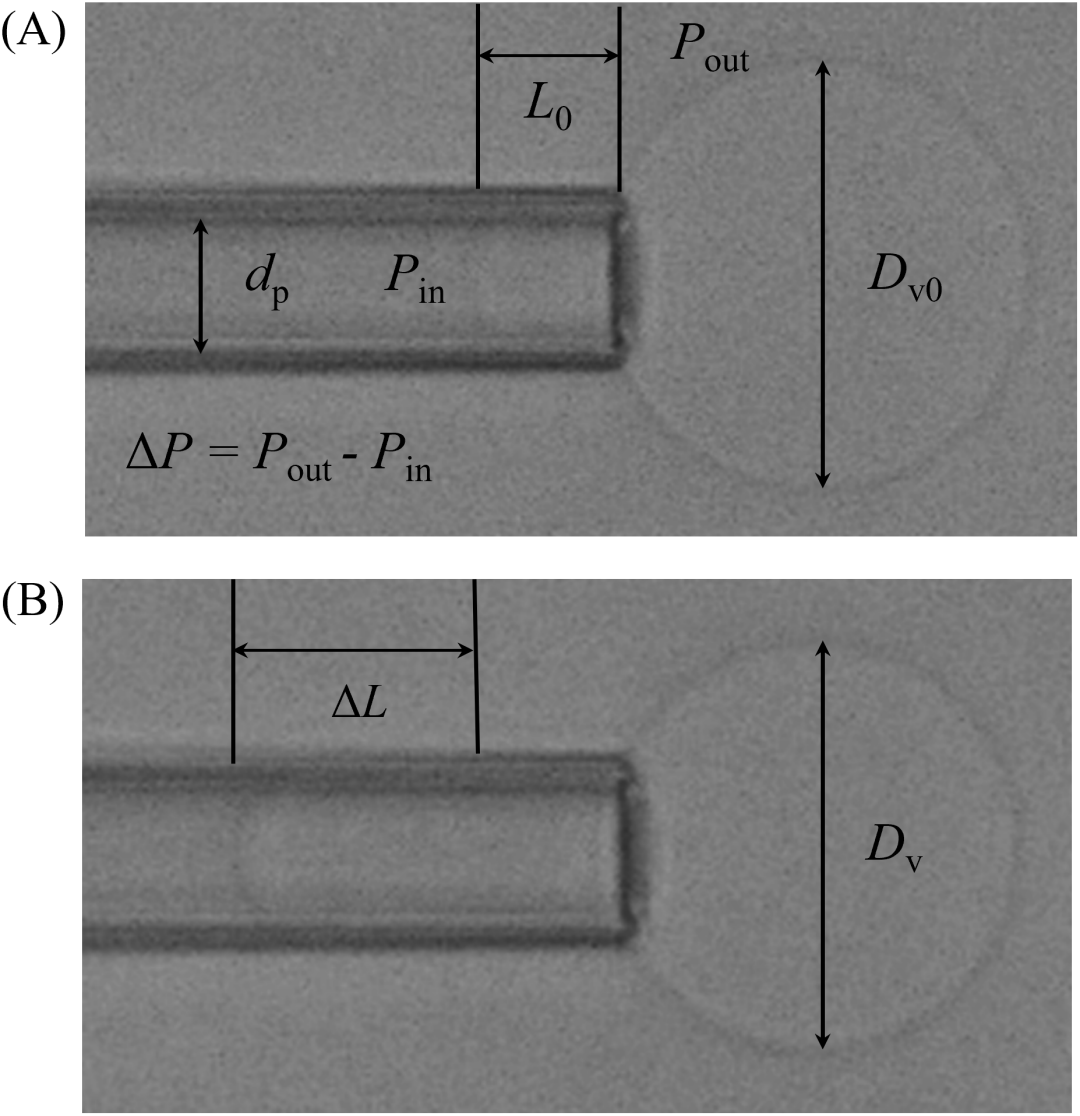
Effect of adsorption of MNPs to a GUV. (A) DIC image of a GUV fixed at the tip of a micropipette using a small aspiration pressure. (B) Change of GUV after adding the solution of MNPs using a micropipette into the suspension of GUVs. The size of GUV was about 30 µm and the internal diameter of the micropipette was about 10 µm.


Micropipette aspiration technique has been performed to measure the fractional area change of a GUV [41,42]. In this case, 200 μL of purified GUVs were taken into the microchamber. Among the many GUVs, a ‘single GUV’ was held at the tip of a micropipette by applying a small holding pressure (~ 1.0 mN/m). Then the GUV remained in a slightly upper position and focused it clearly using DIC of the microscope (Fig 2A). The mechanical tension (*σ*_m_) applied by the micropipette aspiration technique depends on several parameters. In the figure, *D*_v0_ is the diameter of the spherical part of the GUV exterior to the micropipette after applying 1.0 mN/m, *d*_p_ is the internal diameter of the micropipette, and *L*_0_ is the tip length of the GUV after applying 1.0 mN/m. Δ*P* = *P*_out_ − *P*_in_, where *P*_in_ and *P*_out_ are the pressure inside and outside of the GUV, respectively. The applied tension (*σ*_m_) on the membrane of GUV is expressed as follows [34,43]:

$$\sigma_{m}=\frac{\Delta Pd_{p}}{4\left(1-d_{p}/D_{v0}\right)}$$ (2)

Then 40 μL MNPs of concentration 3.0 µg/mL were inserted slowly using a pipette into the suspension, resulting in a final MNPs concentration of 0.5 µg/mL in the microchamber. The time course of the change of GUV was observed and recorded. The fractional change in the area of the GUV membrane is *δ* = Δ*A*/*A*_0_, where *A*_0_ is the area of a GUV before the addition of MNPs and Δ*A* is the change in the area of the GUV membrane after the addition of MNPs. Several parameters are required to obtain *δ* (Fig 2B). One parameter is the change in the projection length, Δ*L* = *L*_eq_- *L*_0_, where *L*_eq_ and *L*_0_ are the projection lengths of the GUV at equilibrium after and before the interaction with MNPs, respectively. The same experiment was repeated with ∼10 GUVs to provide an average value of *δ* and the standard error. The equation for *δ,* assuming constant volume, is given by [34],


$$\delta =\frac{\Delta A}{A_{0}}=\frac{d_{p}\left(1-d_{p}/D_{v}\right)\Delta L}{D_{v0}^{2}}$$ (3)

where *D*_v_ and *D*_v0_ are the diameters of the spherical parts of the GUV at equilibrium after and before the interaction with MNPs, respectively.

## 3. Results

In this section, we have presented the results obtained by the interaction of MNPs with DOPG/DOPC (40/60)-GUVs, DOPC/PEG-DOPE (99/1)-GUVs, and DOPC-GUVs. At first, the deformation of spherical shaped DOPG/DOPC (40/60)-GUVs and DOPC-GUVs are presented. Then the shape changes of prolate (non-axisymmetric), prolate (axisymmetric), fetal (curl), and cylindrical shaped DOPC/PEG-DOPE (99/1)-GUVs have been presented. Lastly, the membrane area changes of the spherical shaped DOPG/DOPC (40/60)-GUVs and DOPC-GUVs are presented.

### 3.1. Deformation and compactness of DOPG/DOPC (40/60)-GUVs and DOPC-GUVs induced by 4.4 µg/mL MNPs

**Fig 3:**
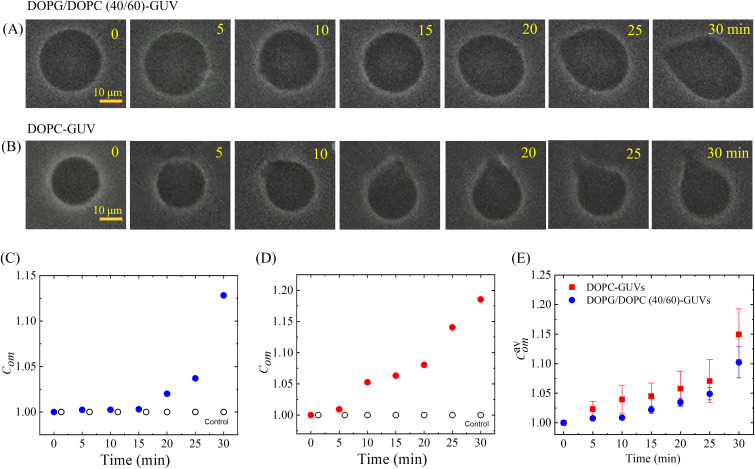
The deformation and compactness (*Com*) of DOPG/DOPC (40/60)-GUVs and DOPC-GUVs induced by 4.4 μg/mL NPs. Phase contrast microscopic images indicate the deformation of a (A) DOPG/DOPC (40/60)-GUV and (B) DOPC-GUV. The number on each image indicates the time in minutes after interacting with MNPs. (C) The time dependent *C*_*om*_ of GUV as presented in (A). (D) The time dependent *C*_*om*_ of GUV as presented in (B). (E) The time dependent average compactness ( $C_{om}^{av}$) of the DOPG/DOPC (40/60)-GUVs and DOPC-GUVs. The data obtained from several independent experiments show the average value with standard error.

At first, we investigated the MNPs-induced deformation of DOPG/DOPC (40/60)-GUV. Fig 3A shows the effects of the interaction of 4.4 μg/mL NPs with a ‘single DOPG/DOPC (40/60)-GUV’. In the absence of MNPs, the GUV exhibits a spherical structure at 0 min. This form does not alter within the first 5 min following the addition of MNPs. A small deformation is initiated at 10 min, while a large deformation is visible from 25 to 30 min. Similar deformation is also observed for several other ‘single GUVs’. This deformation is very similar to that observed in the cholesterol free membranes [22]. Now it has been determined the degree of deformation by measuring its compactness, *C*_*om*_. The values of *C*_*om*_ are 1.0, 1.002, 1.002, 1.003, 1.020, 1.037, and 1.128 at 0, 5, 10, 15, 20, 25, and 30 min, respectively. The time course of *C*_*om*_ of the GUV shown in Fig 3A is presented in Fig 3C. Similarly, the effects of MNPs, at the same concentration, on DOPC-GUV are shown in Fig 3B. The time course of *C*_*om*_ for the GUV depicted in Fig 3B is presented in Fig 3D. Both the GUVs show a similar trend in which *C*_*om*_ increases slowly for the first 25 min and then increases rapidly. The time dependent average compactness ( $C_{om}^{av}$ ) for several DOPG/DOPC (40/60)-GUVs and DOPC-GUVs is shown in Fig 3E. The $C_{om}^{av}$ value for DOPC-GUVs is slightly higher than that of DOPG/DOPC (40/60)-GUVs. The results for the control experiment are presented in Fig 3C, D. The phase contrast images of 5 different DOPG/DOPC (40/60)-GUVs under the same physiological condition of Fig 3A is presented in the Supporting information (S4 in S1 File). The dataset corresponding to Fig 3C–E is shown in S1 Table in S1 File. The compactness measurement procedure is illustrated in S5 in S1 File. These results provide information that the deformation of the GUVs occurred due to the adsorption of MNPs into membranes. Since the added MNPs were mixed with the vesicle suspension, it was not possible to determine how the observed GUVs changed their dynamics, as removing or isolating the MNPs from the vicinity of the GUVs was not feasible.

### 3.2. Shape change of DOPC/PEG-DOPE (99/1)-GUV induced by 1.0 µg/mL MNPs

**Fig 4:**
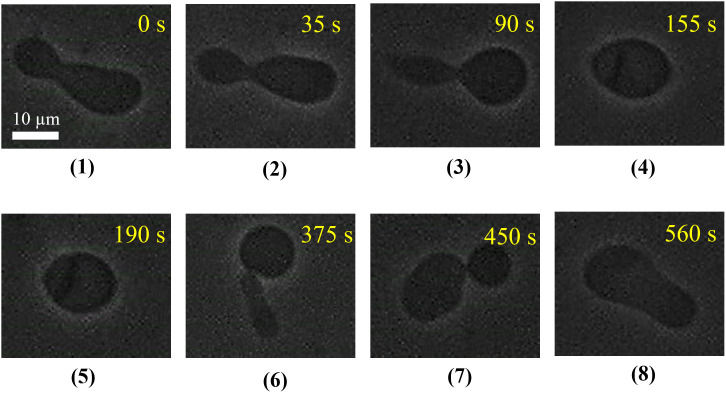
Phase contrast images depict the transformation of the prolate (non-axisymmetric discocyte) shape of DOPC/PEG-DOPE (99/1)-GUV following the addition of 1.0 µg/mL MNPs. The upper panel shows the shape changes after the introduction of MNPs, while the lower panel shows the shapes after the MNPs were removed.

To observe the dynamics of shape change of a GUV induced by MNPs and the reversibility of shape change after stopping the MNPs into the vicinity of GUVs, we utilized the ‘single GUV’ method using the micropipette technique [16]. The solution of MNPs was introduced to the vicinity of a ‘single GUV’ through a glass micropipette and observed its dynamics. Firstly, prolate shape GUV was considered, where the size of the two parts of the prolate remained different. Fig 4 shows a representative experimental result of the shape changes of a DOPC/PEG-DOPE (99/1)-GUV induced by 1.0 µg/mL MNPs. At 0 s, at first in the absence of MNPs, we used a prolate (or non-axisymmetric discocyte shape) as the initial shape of the GUV. After the addition of MNPs, the prolate GUV changed its shape with a smaller neck width at 35 s. Further addition of MNPs, the GUV also contained two parts (one part is nearly spherical and another part is comet type) connected with a narrow neck at 90 s. The shape of the GUV became elliptical at 155 s.

To determine the reversibility of shape changes of the GUV, the addition of MNPs solution through the micropipette was stopped (at 180 s) after the shape change of the GUV was completed. The time course of the reversibility of shape changes of the GUV was observed from 190 s to 560 s. The elliptical shaped GUV changes into two parts, where one part is spherical and another part is cylindrical, connected by a narrow neck at 375 s. Then changed into a non-axisymmetric discocyte shape at 450 s, and finally changed into a prolate shape at 560 s. It has been reasonably considered that after the stopping of NPs, the adsorbed MNPs diffuse away from the external monolayer of the membrane, resulting the GUV regaining its original shape. We observed this shape change in 24 ‘single GUVs’ among 29 examined GUVs (*n *= 29) and in the other five ‘single GUVs’ no shape change is observed under 1.0 µg/mL MNPs. We also investigated similar experiments for several concentrations and obtained the corresponding reversibility. As a control experiment, buffer containing 0.10 M glucose was added under the same conditions, but no shape change of the GUV was observed.

**Fig 5:**
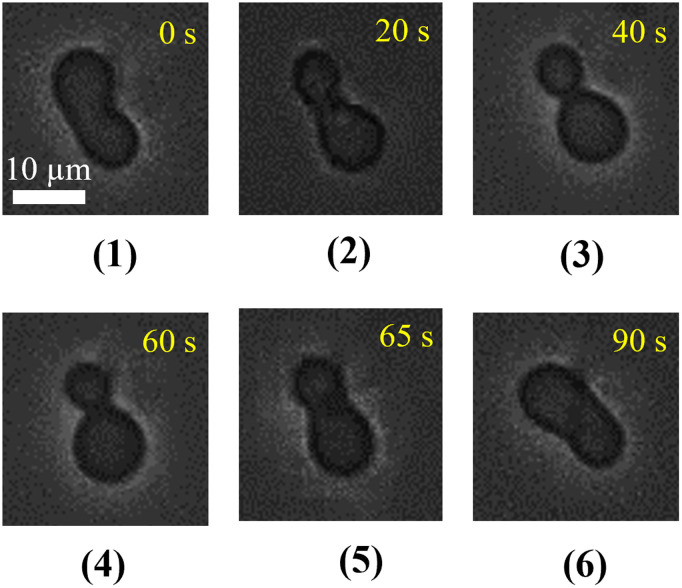
Phase contrast images of the change in prolate (axisymmetric) shape DOPC/PEG-DOPE (99/1)-GUV after addition of 1.0 µg/mL MNPs. The upper and lower panels show the change in shapes after adding and stopping the MNPs, respectively.

Secondly, a prolate (axisymmetric) shaped ‘single GUV’ was considered, where the size of the two parts of the prolate remained almost same. A representative experimental result of the shape changes of a ‘single DOPC/PEG-DOPE (99/1)-GUV’ induced by 1.0 µg/mL MNPs is shown in Fig 5. After the addition of 1.0 µg/mL NPs, the prolate shape changed into a pear at 20 s, then into an asymmetrical two spheres connected by a narrow neck. Finally, the diameter of the neck size reduces with further addition of MNPs as shown in 40 s. As a control experiment, buffer containing 0.10 M glucose was added under the same conditions, but no shape change of the GUV was observed. To determine the reversibility of shape changes of GUV, the addition of MNPs through micropipette was stopped after the shape change of the GUV was completed at 40 s (Fig 5). The time course of the shape changes after the stop of MNPs is presented (e.g., 60–90 s). The two non-spheres connected by a narrow neck change into a pear at 60 s and then change into a prolate shape with relatively higher neck width at 65 s. The prolate shape of GUV appears at 90 s. This is because the MNPs diffuse away from the external monolayer of the GUV into the bulk solution as the concentration of MNPs decreases near the GUV. Therefore, the result of Fig 5 indicates the shape change induced by MNPs was reversible. We observed this shape change in 24 GUVs among 29 examined GUVs (*n *= 29) and in the other five GUVs, their shape remained static and unaltered. We also investigated similar experiments for several concentrations and obtained the corresponding reversibility.

**Fig 6:**
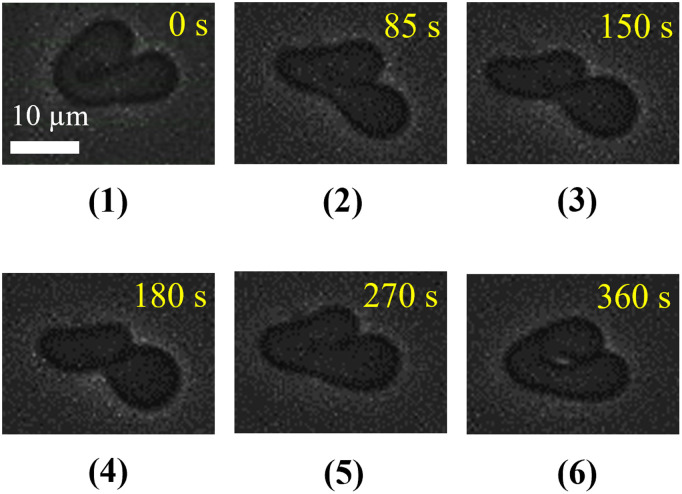
Phase contrast images of the change in fetal-shaped DOPC/PEG-DOPE (99/1)-GUV after addition of 1.0 µg/mL MNPs. The upper panel shows the change in shapes after adding the MNPs. The lower panel shows the change in shapes after stopping the MNPs.

Thirdly, a fetal (curl) shaped ‘single GUV’ prepared by DOPC/PEG-DOPE (99/1) was considered. A representative experimental result of the shape changes of a ‘single GUV’ induced by 1.0 µg/mL MNPs is shown in Fig 6. The lower part of the GUV extended compared to the upper part and the thickness of the neck became smaller at 85 s. The neck became very thin and the fetal fragmented into two parts connected by the neck at 150 s. The addition of MNPs through the micropipette was stopped just after 150 s and the reversibility of the GUV was investigated. The fragmentation of two parts came close and tried to form a thick neck at 180 s. The diameter of the neck became higher at 270 s and the GUV shows a similar shape as observed at 85 s. The reversibility of shape change was completed at 360 s and the shape of the GUV looks similar as observed in 0 s. A similar transformation of the changes in shape was observed for several GUVs (*n *= 24).

**Fig 7:**
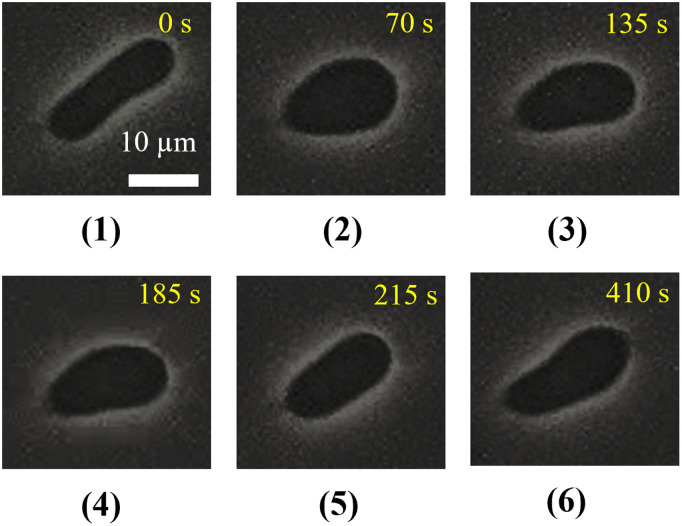
Phase contrast images showing the transformation of the cylindrical-shaped DOPC/PEG-DOPE (99/1)-GUV after the addition of 1.0 µg/mL MNPs. The upper panel depicts shape changes following the introduction of MNPs, while the lower panel illustrates shape changes after the MNPs were removed.

Finally, a cylindrical-shaped ‘single GUV’ prepared from DOPC/PEG-DOPE (99/1) was examined. A representative experimental result illustrating the shape changes of the GUV induced by 1.0 µg/mL MNPs is shown in Fig 7. After the addition of MNPs the GUV transformed into an ovoid (balloon) shape within 70 s. Then the central region of the GUV became slightly narrowed and the GUV changed into an ellipsoidal shape at 135 s. To observe the reversibility of these changes, the MNPs injection was stopped after 135 s. The central region of the GUV appeared flattened at 185 s as the GUV began to form a prolate shape. After further observation, it was revealed that by 410 s the GUV almost regained its original cylindrical shape. A similar reformation of the GUV shapes was also observed in several experiments (*n *= 18).

### 3.3. Fraction of shape change, reform, and non-reform of DOPC/PEG-DOPE (99/1)-GUVs induced by MNPs

**Fig 8:**
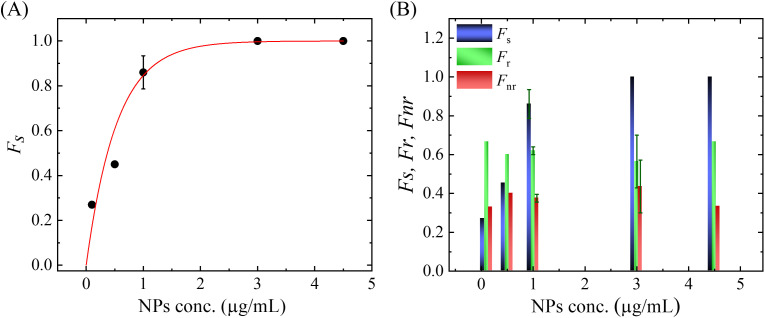
Fraction of shape changed, reformed, and not reformed of DOPC/PEG-DOPE (99/1)-GUVs induced by MNPs. (A) Dependence of the fraction of shape changed GUVs on NPs concentration. (B) Comparison among the fraction of shape change, reformed, and not reformed of GUVs for various concentrations of MNPs.


Now we present various types of results under different MNPs concentrations. Fig 8. indicates the interaction of various concentrations of MNPs with DOPC/PEG-DOPE (99/1)-GUVs (observed 7–12 ‘single GUVs’ in each MNPs concentration). Here, the threshold concentration of MNPs is defined as the concentration at which 50% of the observed GUVs induced a shape change [37]. According to these results, the threshold concentration of MNPs is 0.5 µg/mL. As the MNPs concentration increases, the fraction of shape changed GUVs (*F*_s_) increases as shown in Fig 8A. At concentration≥3.0 µg/mL, the change in shape of GUVs becomes 100%. The MNPs concentration (*C*) dependent *F*_s_ is well fitted to a single exponential growth function [44],

$$F_{s}=1-\exp\left(-k_{s}C\right)$$ (4)

where *k*_s_ is the rate of shape change over MNPs concentration. From the fitting, the value of *k*_s_ is obtained as 0.533 (μg/mL)^-1^.

The fraction of shape changes with reversibility (*F*_r_) and non-reversibility (*F*_nr_) of GUVs is presented in Fig 8B. The value of *F*_r_ is about twice that of *F*_nr_ for all concentrations of MNPs. It means that about 33% of GUVs did not reverse to their original shape after applying the MNPs in the suspension. The dataset for *F*_s_, *F*_r_, and *F*_nr_ is presented in the S3 Table in S1 File.

The changes in shape, as observed in Figs 4–7, were reversible after stopping the MNPs. From these investigations, it can be reasonably considered that MNPs adsorbed to the outer monolayer of the membranes, and a mismatch between the area of outer monolayer and inner monolayer occurred, which induced the shape change of the GUVs. After stopping the MNPs, the NPs in the vicinity of the GUVs diffused to the entire suspension and then reverted to the original shape. However, it is not possible to understand whether the MNPs bind the outer monolayer or both monolayers from these investigations. Therefore, it is necessary to investigate the change in fractional area of the membrane after interacting the MNPs into the GUV. In this regard, we utilized the well-known micropipette aspiration technique. In this technique, a ‘single GUV’ held at the tip of a micropipette using a small suction pressure, e.g., membrane tension 1.0 mN/m, and MNPs applied to the vesicle suspension. The GUV can be aspirated into the micropipette if a nanopore is formed in the membranes [32,45].

### 3.4. Membrane area change of the DOPC-GUV and DOPG/DOPC (40/60)-GUV induced by MNPs

**Fig 9:**
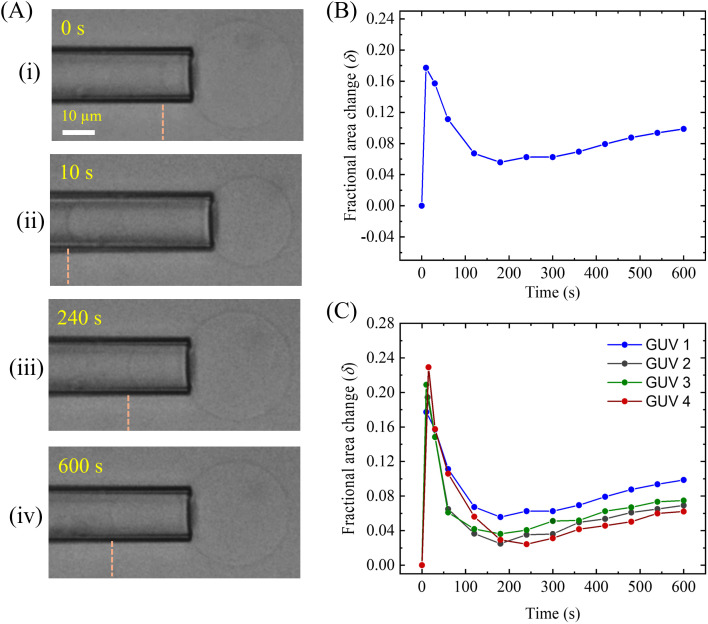
The fractional change in the area ( *δ*) of the DOPC-GUVs induced by 0.5 μg/mL MNPs. (A) Change in tip length and the size of the GUV at (i) 0 s, (ii) 10 s, (iii) 240 s, and (iv) 600 s. (B) Time course of the change in *δ* for the GUV as shown in (A). (C) Time course of the change in *δ* for several DOPC-GUVs under the same condition of (A).

This section focuses on the fractional change in the area (*δ*) of a membrane induced by 0.5 μg/mL MNPs. Fig 9 shows the time course of the change of *δ* of DOPC-GUV. A representative experimental result for DOPC-GUV after inducing the MNPs is shown in Fig 9. The dynamic change in the tip length and the size of GUV at different times is presented in Fig 9A. The tip length at first increases and then decreases and finally again increases. The time course of the change in *δ* of the GUV shown in Fig 9A is presented in Fig 9B. There are three regimes of the change of *δ* with time. In the first stage, after adding the MNPs into the GUV, the *δ* increases from 0 with time very rapidly and reaches a maximum value of 0.18 by 10 s. In the second stage, the *δ* decreases from 0.18 to 0.06. This decrement takes about 180–10 = 170 s. In the third stage, the *δ* again increases slightly, e.g., from 0.06 to 0.10 for a time duration of 600–180 = 420 s. The similar trend of the changes of *δ* was obtained in GUVs under the same experimental condition (Fig 9C). From Fig 9C, the maximum value of *δ* varies from 0.18 to 0.23 (average 0.2) within 10–15 s, the lower value of *δ* varies from 0.02 to 0.06 (average 0.03) within 180–240 s, and at 600 s the value of *δ* varies from 0.1 to 0.06 (average 0.08). A control experiment using the buffer solution instead of MNPs solution was also employed and could not obtain the changes of *δ* over time. The dataset for *δ* of DOPC-GUVs is presented in the S4 Table in S1 File.

**Fig 10:**
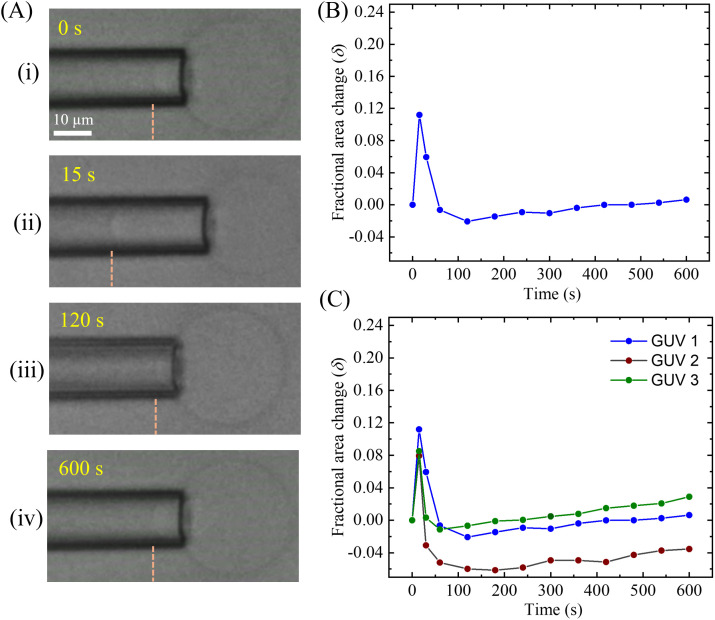
The fractional change in the area (*δ*) of the DOPG/DOPC (40/60)-GUVs induced by 0.5 μg/mL MNPs. (A) Change in tip length and the size of the GUV at (i) 0 s, (ii) 15 s, (iii) 120 s, and (iv) 600 s. (B) Time course of the change in *δ* for the GUV as shown in (A). (C) Time course of the change in *δ* for several DOPG/DOPC (40/60)-GUVs under the same condition of (A).

We performed the similar experiment for DOPG/DOPC (40/60)-GUV and observed the changes in *δ* with time after inducing the 0.5 μg/mL MNPs (Fig 10). The dynamic change in the tip length and the size of a ‘single GUV’ at different times is presented in Fig 10A. The tip length at first increases and then decreases and finally again increases. The time course of the change in *δ* of the GUV shown in Fig 10A is presented in Fig 10B. In this case, we also observed the similar trend of the change of *δ* with time. At first, increases the *δ* with time, then decreases to negative value and finally a small increase over time. The time to increase the *δ* at maximum value (0.11) is about 15 s. Then the value of *δ* decreases to −0.02 for a time duration of 120−15 = 105 s. The *δ* further increases to 0.01 over a longer period. The similar trend of the changes of *δ* was obtained in several ‘single GUVs’ under the same experimental condition (Fig 10C). The only difference between the two types of GUVs is the maximum value of *δ*, DOPC-GUVs shows more *δ* than DOPG/DOPC (40/60)-GUVs induced by the same concentration of MNPs (see Figs 9C and 10C). In this investigation, during the initial rapid increase, then decrease, and finally a small increase of *δ*, we could not observe the aspiration of GUV into the micropipette using 0.5 μg/mL. Similar results were also observed for many ‘single GUVs’ in several independent experiments. The dataset for *δ* of DOPG/DOPC (40/60)-GUVs is presented in the S4 Table in S1 File.

**Fig 11:**
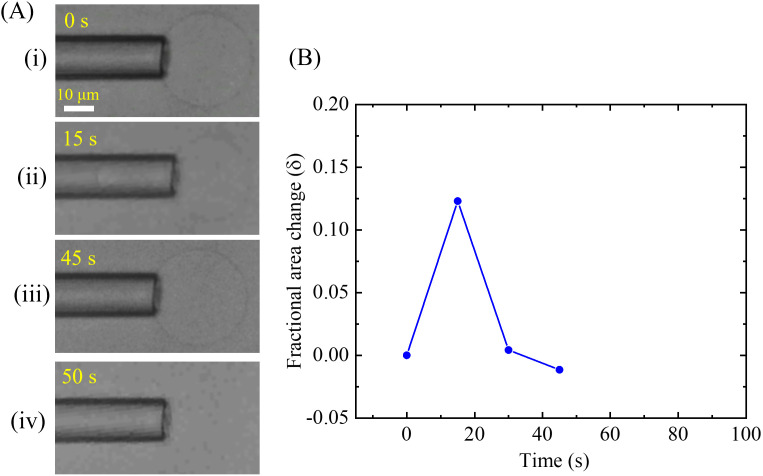
The fractional change in the area (*δ*) of the DOPG/DOPC (40/60)-GUV induced by 0.8 μg/mL MNPs. (A) Change in tip length and the size of the GUV at (i) 0 s, (ii) 15 s, (iii) 45 s, and (iv) 50 s. (B) Time course of the change in *δ* for the GUV as shown in (A).

In the next step, a higher concentration of MNPs was used, e.g., 0.8 μg/mL in the DOPG/DOPC (40/60)-GUV and observed the changes in *δ* with time. An experimental representation is shown in Fig 11. The dynamic change in the tip length and the size of GUV at different times is presented in Fig 11A. The tip length at first increases and then decreases and finally GUV aspirated into the micropipette. The time course of the change in *δ* of the GUV shown in Fig 11A is presented in Fig 11B. At first, increases the *δ* with time, then decreases to negative value and GUV aspirated. The time to increase the *δ* at maximum value (0.123) is about 15 s. Then the value of *δ* decreases to −0.011 for a time duration of 45−15 = 30 s. There was no further increase in *δ* as the GUV was aspirated into the micropipette.

## 4. Discussion

We found that when 4.4 µg/mL MNPs were introduced into the suspension of vesicles, the deformation of GUV was observed (Fig 3). The prolate (two types of prolate), fetal and cylindrical shaped GUVs changed their shape into different types after introducing the 1.0 µg/mL MNPs and later the GUVs exhibited the reversibility after stopping the MNPs (Figs 4–7). The fraction change in shape increases with the increase of MNPs concentration (Fig 8). Using the micropipette aspiration technique, we found that the fraction change in area (*δ*) of a GUV followed three regimes after adding the 0.5 µg/mL MNPs. At first, the *δ* increases with time and reaches a maximum value within 10–15 s, then decreases the *δ* to almost 0, and finally, the *δ* increases slightly over time. A similar trend is observed for several independent experiments over many ‘single GUVs’.

The headgroup of DOPC contains a (P^−^−N^+^) dipole [22,26,46] that is tilted at an angle of 13° relative to the membrane surface [47]. Anionic MNPs interact with the N^+^ group via Coulombic attraction (electrostatic forces), leading to a change in the tilt angle. At the same time, electrostatic repulsion occurs between the MNPs and the DOPG molecules, as well as between the MNPs and the P^−^ group, and contributes to vesicle deformation and membrane poration. Furthermore, the nonpolar regions of lipid molecules interact with MNPs through hydrophobic interactions and van der Waals force [48]. As a result, the interaction between anionic MNPs and DOPG/DOPC membranes is controlled by a combination of electrostatic forces, van der Waals attractions, and hydrophobic interactions.

The shape change of vesicles can be determined by the area difference elasticity (ADE) model [49,50]. In this model, the area of each monolayer is not fixed at its equilibrium value but can stretch elastically to increase the bilayer’s nonlocal elastic energy. Therefore, the total elastic energy of the GUV (*W*_el_) is the sum of the membrane bending energy (*W*_b_) and the relative monolayer stretching energy (*W*_r_) which is given by the following expression,


$$W_{el}=W_{b}+W_{r}=\frac{1}{2}k_{c}\int {\left(C_{1}+C_{2}\right)}^{2}dA+\frac{1}{2}\frac{k_{r}}{A_{0}h^{2}}\left(\Delta A-\Delta A_{0}\right),$$ (5)

where *k*_c_ and *k*_r_ are the local and nonlocal bending modulus, respectively. Here, $\Delta A_{0}(={A_{0}}^{out}-{A_{0}}^{in})$ is the area difference between the outer and inner monolayers in the non-stretched state (i.e., in the absence of MNPs), $\Delta A(=A^{out}-A^{in})$ is the area difference the two monolayers in the stretched state (i.e., in the presence of MNPs) and h is the gap between the neutral surfaces of the two monolayers.

In the ADE model, the shape of the GUV is determined by the minimization of the elastic energy $\left(W_{el}\right)$ for a given area *A*, volume *V*, and area difference $\Delta A_{0}$. While the volume is constant, the deviation of the vesicles from spherical shape is governed by the term $(\Delta A-\Delta A_{0})²$. As a result, the adsorption of MNPs onto the membrane progressively increases the degree of deformation (i.e., compactness) over time (Fig 3). During interaction with MNPs, if the free energy of a deformed GUV (*E*_def_) exceeds that of a spherical GUV (*E*_sph_) such that *E*_def_>> *E*_sph,_ the GUV deviates from its original spherical shape and undergoes deformation. The fraction of deformation is ${Fr}_{d}\approx \frac{1}{E_{def}}(1+\frac{E_{sph}}{E_{def}})$ [22].

The experimental results clearly indicate that even a very low concentration (1.0 μg/mL) of MNPs can induce several types of shape changes in DOPC/PEG-DOPE (99/1)-GUVs. During the experiment, a solution of MNPs at 1.0 μg/mL was gradually added near a ‘single GUV’ using a micropipette over a period of less than 10 min. As MNPs were introduced, some MNPs diffused from the area near the GUV into the bulk phase. Therefore, the observed events occurred under steady-state ion concentration near the GUV, not at equilibrium. The ion concentration near the GUV in this steady-state condition was slightly lower than that of the solution added via the micropipette. Furthermore, within a very short time of MNPs addition, equilibrium binding of the MNPs to the membrane surface may not have been achieved. Thus, it is important to note that the observed effects of MNPs on GUV shape occurred under steady-state conditions and for less than 10 min. When the addition of MNPs was stopped, the ions near the GUV diffused into the bulk phase, causing the ion concentration near the GUV to gradually decrease to 0 μg/mL. During this dilution process, it was observed that the GUV shapes returned to their original forms, indicating that below the critical concentration of MNPs, the shape changes in GUVs were reversible. What effects do these ions have on the phospholipid membranes to induce such shape changes in the GUVs? To address this, it is important to analyze the effects of MNPs on the fractional change in area (*δ*) as well as the membrane’s physical properties.

Now we discuss the change in *δ* with time induced by MNPs. The behavior of the micropipette tip during the interaction of anionic MNPs with DOPG/DOPC (40/60)-GUV and DOPC-GUV likely reflects complex biophysical processes. The possible mechanism can be interpreted as follows. When the anionic MNPs first interact with the PG/PC membrane, it binds to the outer monolayer of the membrane surface, particularly to the positively charged N^+^ group in DOPC. The electrostatic repulsive forces between the MNPs and DOPG as well as between the MNPs and P^−^, do exist and play a role in the change in *δ*. In addition, the chain (e.g., nonpolar part) of the lipid molecules interacts with the MNPs through van der Waals interactions and hydrophobic effects [48]. Thus, the interaction between MNPs and DOPG/DOPC (40/60)-GUV is primarily governed by a balance of electrostatic, van der Waals, and hydrophobic effects. This binding of MNPs may initially stretch the outer monolayer of the membranes, resulting in the stretching in the inner monolayer. Thus, the tension at the contact point, causing the micropipette tip to appear larger due to localized swelling or deformation in response to the increased tension. A suction pressure (1.0 mN/m) was applied to the GUV using the micropipette, which caused the membrane to stretch more and elongate into the pipette, leading to an initial increase in tip length (e.g., *δ*). For fluid bilayer, the elastic energy [51], $E=\frac{1}{2}K_{a}{\left(a-a_{0}\right)}^{2}/a$, where *a* is the area of a lipid molecule, *a*_0_ is the optimal head group area of a lipid molecule, and *K*_a_ is the area compressibility modulus of the bilayer. Thus, electrostatic interaction and membrane deformation might be the possible reasons for the increase of *δ* over time. It is well reported that the *δ* increased due to the stretching of membranes while the antimicrobial peptide, magainin 2 interacted with the lipid membranes of DOPG/DOPC (40/60)-GUV [32,45]. The expansion of the surface area of the lipid membranes was investigated by the interaction of AuNPs of size from 5 to 30 nm. This occurred due to the increase in fluidity of DMPC liposomes [52], resulting in shape changes of the liposomes. It is hypothesized that the localized stress in lipids, caused by electrostatically adsorbed AuNPs, drives the dominant long-range effect of membrane fluidization.

After this initial interaction, the MNPs may induce changes in the membrane structure. For example, the anionic MNPs could cause charge redistribution, leading to a relaxation of tension in the membrane. It has been reported that the surface reconstruction of phospholipid membranes is induced by NPs. The negatively charged carboxyl-modified polystyrene latex with a diameter of 20 nm NPs induces local gelation in fluid bilayers, while positively charged amidine-modified of the same NPs cause locally gelled membranes to fluidize [53]. This may result in a reduction in membrane tension, leading to a decrease in the micropipette tip size as the membrane compressed and accommodated the MNPs. Membrane stiffening occurs as the bound MNPs restrict the fluidity of the membrane lipids. The increased local rigidity means the membrane is less able to stretch, resulting in a decrease in *δ*. The accumulation of many anionic MNPs on the membrane surface can generate electrostatic repulsion between the bound particles. This extra repulsive force resists further MNPs binding and may cause the membrane to retract slightly as it adopts a more energetically stable configuration.

As the interaction progresses, additional factors like MNPs adsorption to deeper of outer monolayer or membrane reorganization could come into play. The final increase in the micropipette tip might be caused by the continued MNP adsorption, which increases the membrane’s overall surface tension. In this stage, the membrane could stretch or deform slightly due to the accumulation of MNPs, resulting in another increase in the *δ*. Thus, the sequence of changes of *δ* likely reflects a dynamic balance between the forces exerted by the MNPs (such as Coulombic and van der Waals interactions) and the inherent mechanical properties of the membrane (such as tension and elasticity).

The fractional area increase has been investigated while neurosteroid, allopregnanolone (3α,5α-tetrahydroprogesterone or Allo) of a 100 nM solution interacted with DOPC/bSM/chol (1:1:1)-GUVs using the micropipette method. Additionally, the author group also investigated an area decrease followed by the usual increase for the same GUVs induced by Allo solution. In contrast, of one of the isoforms of Allo, isoallopregnanolone (3β,5α-tetrahydroprogesterone or isoAllo) decreases the lipid bilayer area and when applied at the same nanomolar concentrations the GUVs [33].

As we stated in section 3.4, the GUV is aspirated into the micropipette if a nanopore is formed in the membranes [42,54]. Based on these investigations, we can consider that during the changes of the tip of the micropipette, the pore was not formed in the membranes of GUVs because we could not observe aspiration of GUVs. In addition, the MNPs bind only in the outer monolayer of the membranes, and those MNPs were not transferred to the inner monolayer before pore formation in the membranes. The mechanism is quite similar to the antimicrobial peptide induced membrane poration in the membranes [32,45]. Once a nanopore is formed in the membranes, the MNPs can diffuse to the inner monolayer of the membrane along with the inside of vesicles [13,55].

The change in compactness over time, as shown in Fig 3, results from the combined effects of the three regimes of *δ* variation over time depicted in Figs 9 and 10. The fractional area change exhibits distinct transient stages: initial stretching, relaxation, and subsequent re-deformation. Initially, the fractional area of GUVs increases by 10–20% within 15 s, then decreases due to membrane stiffening. Finally, the area increases again slightly, by approximately 1–10%, because of the continuous adsorption of MNPs. A combination of these three stages is also observed in Fig 4, where GUV deformation increases (~12–15%) over time due to interactions with MNPs.

## 5. Conclusions

MNPs have become a focus of extensive research due to their promising applications in drug delivery, MRI, hyperthermia cancer therapy, and biosensing. Despite the potential uses of MNPs in different areas, concerns about their mechanism of interaction with biological systems are significant. This paper investigates the dynamics of a ‘single GUV’ induced by anionic MNPs prepared using the green synthesis method. The deformation of a ‘single GUV’ induced by MNPs increased over time. Shape transformations, such as the transition from a prolate to two spheres connected by a neck and from a sphere to two small vesicles connected at a point, were observed when MNPs were introduced in the vicinity of the GUVs. The reversibility of these shape transformations was also observed after the MNPs were removed. The fraction of shape changes increased with the rise in MNPs concentration. The membrane area initially increased rapidly, then slowly decreased back to its original state, and finally increased very slowly over time after the introduction of MNPs into the GUV. These results reflect rapid stretching, followed by slower compression, and finally a slow stretching of the membranes. As MNPs hold great promise for medical and industrial applications, understanding the dynamics of their adsorption into the membranes of a single vesicle or single cell will be a significant advancement for appropriate design and safety considerations.

## Supporting information

S1 File Supporting Information (S). (PDF)
