# Supplementary material for: Exploring the dynamics of a single vesicle induced by Fe₃O₄ nanoparticles using micropipette manipulation
Source: PLoS One. 2025 Jul 7;20(7):e0327639. doi: 10.1371/journal.pone.0327639 (PMC12233257; doi:10.1371/journal.pone.0327639)
Supplement: S1 File — (PDF) [file pone.0327639.s001.pdf]

Supporting Information (S)

Exploring the dynamics of a single vesicle induced by Fe_3O_4 nanoparticles using micropipette manipulation

Nazia Ahmed[1,2], Tawfika Nasrin[1], and Mohammad Abu Sayem Karal[1]*

[1]Department of Physics, Bangladesh University of Engineering and Technology, Dhaka 1000, Bangladesh
[2]Department of Mathematical and Physical Sciences, East West University, Dhaka 1212, Bangladesh

S1 Synthesis of MNPs

The green synthesis method was employed to synthesize MNPs mediated by *Ipomoea Aquatica* leaf extracts. [1]. The detailed description of the characterization is described in our previous paper [1,2]. The MNPs were synthesized by our group, and we have published a series of studies on them over the past few years [3–7]. In this paper, as well as in all our previous publications, we used the same MNPs to ensure consistency in our results. Moreover, we conducted several control experiments (e.g., compactness measurement, fraction of deformation, and fraction of poration) to assess the stability of the MNPs. Briefly, at first, fresh green leaves of *Ipomoea Aquatica* were collected and properly washed with MilliQ water (S1A Fig.). Then a fine paste was made by drying the leaves (S1B Fig.). A 60 g paste was mixed into 0.40 L deionized water and kept at 80 °C for 4 hours at 800 rpm. This solution was then filtered, cooled down to room temperature, and collected as leaf extract. The biomolecules of leaf extracts were used for reducing and capping agents of MNPs. Now 20 mL of both 5 mM $FeCl_2.4H_2O$ and 10 mM $FeCl_3$ were diluted together by maintaining the temperature at 60 °C under 800 rpm and after 10 min, an amount of 5 mL leaf extracts was added into the mixture. After another 10 min, 100 mL 10 mM NaOH was poured into the solution of the mixture to form the MNPs colloidal solution. Now the precipitated MNPs were collected from the solution by using a bar magnet, dried at 60 °C for a few days, and then ground into fine powder.

The overall reaction is given by,

Ipomoea Aquatica + $FeCl_2 \cdot 4H_2O$ (aq) + $2FeCl_3$ (aq) + 8NaOH (aq) → [*Ipomoea Aquatica* / Fe_3O_4] ↓(s) + 8NaCl + $8H_2O$ (aq)

These MNPs were diluted in the DEG and buffer solution to prepare a 0.025 mg/mL MNPs solution (stock). From this stock solution, various concentrations (e.g., 0.1, 0.5, 1.0, 3.0, 4.4, 4.5 µg/mL) of MNPs solution were prepared for further applications. Here, the synthesized MNPs exhibited a

cubic inverse spinel structure [1], whose size was 18 nm (S1C Fig.) with a zeta potential of −21.3 mV (S1D Fig.).

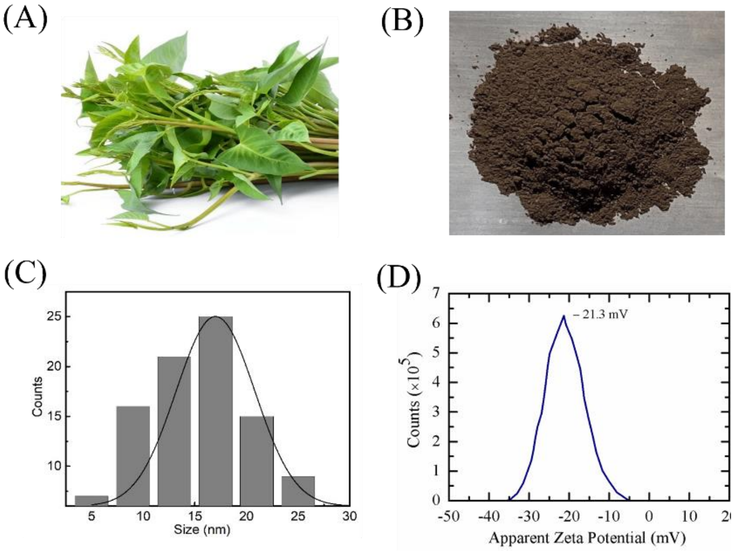

S1 Fig. Synthesis and characterization of MNPs. (A) *Ipomoea Aquatica* leaf. (B) Fine powder of MNPs. (C) Average particle size by dynamic light scattering (DLS). (D) Zeta potential of MNPs. The (C) has been adapted from ref. [1] with permission, and (D) has been adapted from ref. [2] with permission.

S2 UV-Vis spectroscopy of the synthesized MNPs

We performed UV-Vis spectroscopy on samples at concentrations of 0.1, 0.5, 1.0, and 4.5 µg/mL in PIPES buffer (S2 Fig.) to assess whether the size of the Fe_3O_4 nanoparticles (MNPs) varies with concentration. In all cases, the absorbance spectra exhibited a peak around 214 nm, suggesting that the size of the MNPs remained consistent across different concentrations. This peak position is consistent with previous studies on MNPs, which reported absorbance peaks at 215 nm for particles sized 15.5 nm [8], and at 229 nm for particles sized 65 nm [9]. Given the very low concentrations of MNPs used, the likelihood of aggregation is negligible, further supporting the observation that the nanoparticle size remained unchanged (as shown in S2 Fig.). It is well established that nanoparticle size can vary depending on the synthesis method and conditions [10–12]. In our study, we initially synthesized MNPs at a higher concentration using a green synthesis technique and subsequently diluted the solution to obtain lower concentrations. Since the synthesis conditions were identical for all concentrations, the resulting MNPs maintained the same size within the experimental error.

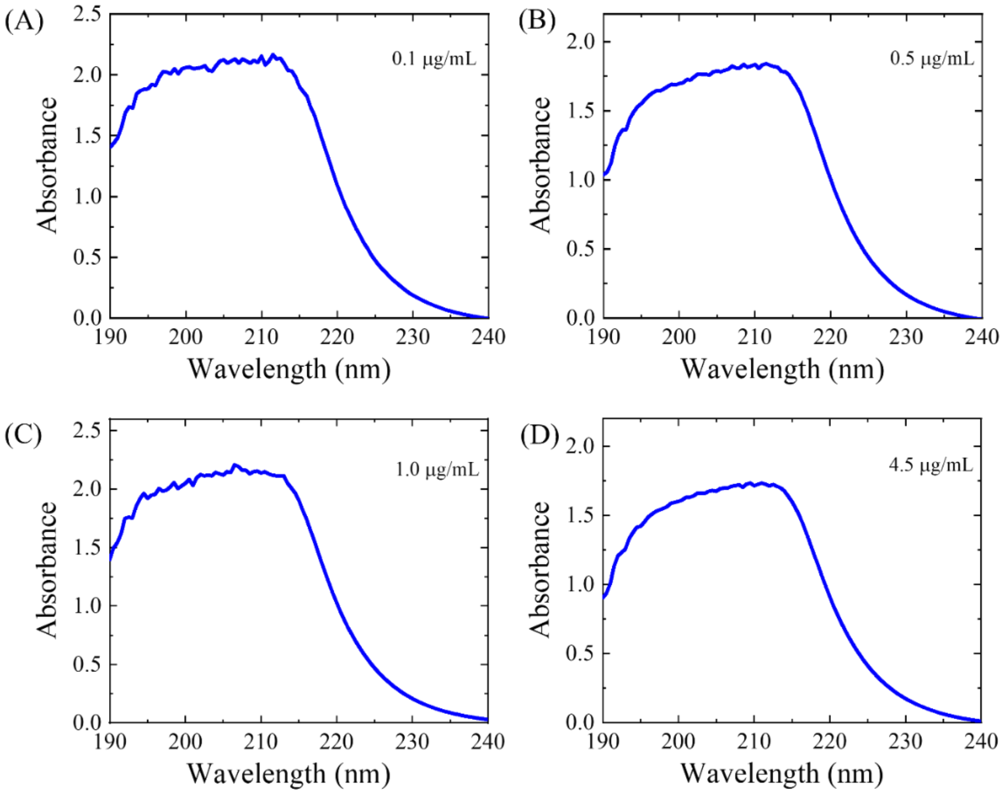

S2 Fig. UV-Vis spectroscopy of the synthesized MNPs at concentrations of (A) 0.1, (B) 0.5, (C) 1.0, and (D) 4.5 μg/mL.

S3 Microchamber

The microchamber, as shown in S3 Fig., comprised a glass slide, a silicon-rubber spacer, and a cover slip, forming a U-shaped structure. The microchamber was positioned on the thermo-controlled stage of the microscope.

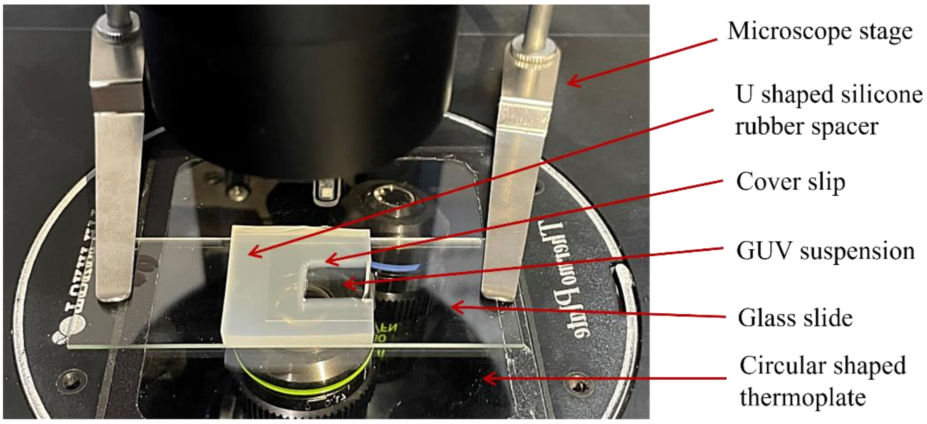

S3 Fig. A photo of microchamber used for the observations of GUVs.

S4 Deformation of several of DOPG/DOPC (40/60)-GUVs induced by 4.4 µg/mL MNPs

Here, we investigated the MNPs-induced deformation of several DOPG/DOPC (40/60)-GUVs under the same condition as presented the results in Section 3.1. S4(A-E) Fig. shows the effects of the interaction of 4.4 µg/mL MNPs with 5 different GUVs. In the absence of MNPs, all the GUVs exhibit a spherical structure at 0 min. This form does not alter within the first 5 min following the addition of MNPs. A small deformation is initiated at 10 min, while a large deformation is visible from 25 to 30 min for all GUVs.

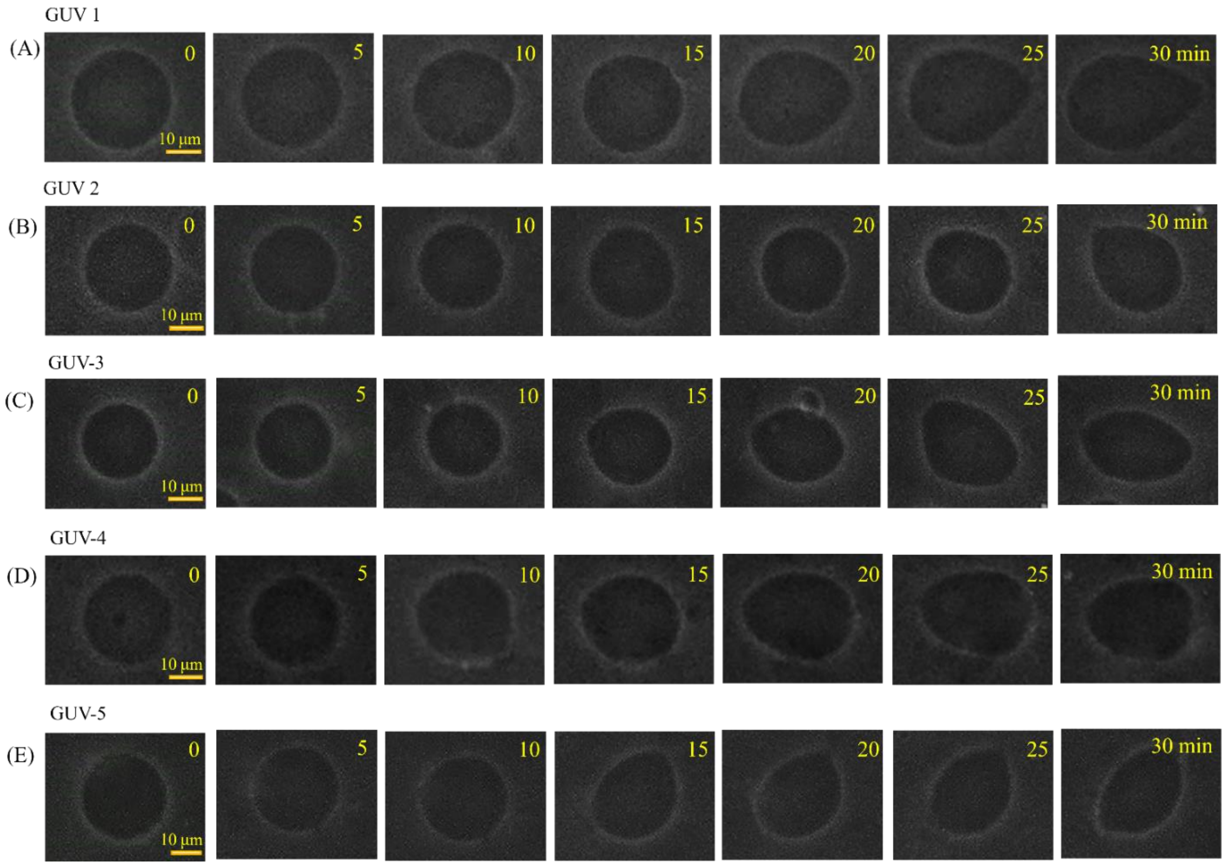

S4 Fig. The deformation of several DOPG/DOPC (40/60)-GUVs induced by 4.4 µg/mL NPs. (A-E) Phase contrast microscopic images indicate the deformation of five different GUVs under the same physiological condition. The number on each image indicates the time in minutes after interacting with MNPs.

The degree of deformation is determined by measuring its compactness (C_{om}). The time course of C_{om} of these GUVs along with control experiment is shown in S5 Fig. All the GUVs show a similar trend in which C_{om} increases slowly for the first 25 min and then increases rapidly. The time dependent values of C_{om} for these GUVs are shown in Table S2.

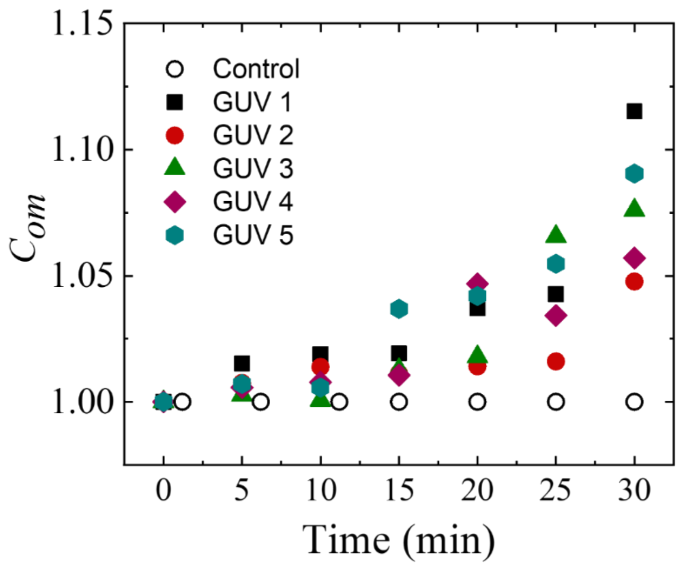

S5 Fig. The compactness (C_{om}) of several DOPG/DOPC (40/60)-GUVs induced by 4.4 μg/mL NPs. The time dependent C_{om} of 5 different GUVs as presented in S3(A-E).

S5 Compactness measurement

S6 Fig. presents an illustration of the compactness (C_{om}) measurement for both a perfectly spherical-shaped GUV and a deformed GUV. The analysis involved selecting the rim of the images, highlighted by a dark red dotted line, and extracting the corresponding perimeter (P) and cross-sectional area (S_{cr}) of the GUV using MATLAB's Image Processing Toolbox. In S6(A) Fig., the obtained C_{om} value is 1.0, indicating a perfect spherical shape, whereas S6(B) Fig. shows a higher C_{om} value, signifying a deformed shape. The parameter C_{om} can be related to the physical parameter such as surface area (S_t) of the deformed GUV at time t as follows [2]:

$$S_t \approx S_0[1 + 1.78\sqrt{(C_{om} - 1)}] \quad \text{(S1)}$$

where S_0 surface area of the spherical-shaped GUV before the interaction of MNPs. Both C_{om} and S_t/S_0 increases with time as obtained in our previous investigation [2].

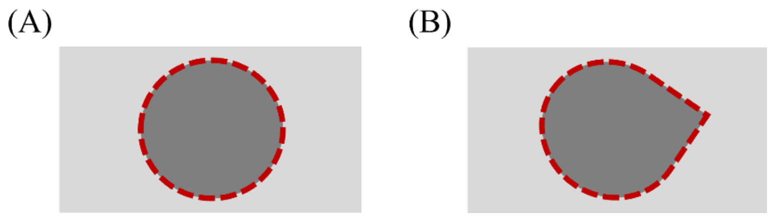

S6 Fig. Illustration of measuring the compactness of a 'single GUV'. (A) A perfectly spherical shaped GUV. (B) Deformed GUV

S1 Table. Time dependent compactness (C_{om}) and average compactness (C_{om}^{av}) of DOPG/DOPC (40/60)-GUVs and DOPC-GUVs corresponding to Fig 3(C-E).

Time (min)	DOPG/DOPC (40/60)	DOPC	DOPG/DOPC (40/60)		DOPC	
	C_{om}	C_{om}	C_{om}^{av}	±SErr	C_{om}^{av}	±SErr
0	1	1	1	0	1	0
5	1.00233	1.00926	1.00751	0.00148	1.02323	0.01299
10	1.00243	1.05274	1.00875	0.00204	1.03953	0.02319
15	1.00304	1.06326	1.02209	0.00624	1.04498	0.02243
20	1.02010	1.08059	1.03486	0.00697	1.05781	0.02906
25	1.03705	1.14071	1.04901	0.01036	1.07068	0.03617
30	1.12831	1.18551	1.10218	0.02619	1.14966	0.04310

S2 Table. Time dependent compactness (C_{om}) of DOPG/DOPC (40/60)-GUVs corresponding to S5 Fig.

Time (min)	GUV 1	GUV 2	GUV 3	GUV 4	GUV 5
	C_{om}	C_{om}	C_{om}	C_{om}	C_{om}
0	1	1	1	1	1
5	1.01520	1.00751	1.00264	1.00565	1.00711
10	1.01885	1.01382	1.00063	1.00776	1.00564
15	1.01921	1.01127	1.01273	1.01055	1.03685
20	1.03705	1.01407	1.01785	1.04680	1.04195
25	1.04263	1.01603	1.06556	1.03423	1.05475
30	1.11518	1.04770	1.07599	1.05703	1.09049

S3 Table. Nanoparticle's concentration depending on the fraction of shape changed GUVs (F_s), fraction of shape changed with reversibility (F_r), and non-reversibility (F_{nr}) of DOPC/PEG-DOPE (99/1)-GUVs corresponding to Fig 8(B).

Conc. (µg/mL)	F_s		F_r		F_{nr}	
	Average	±SErr	Average	±SErr	Average	±SErr
0.1	0.27	0	0.667	0	0.33	0
0.5	0.454	0	0.6	0	0.4	0
1.0	0.861	0.074	0.62	0.02	0.376	0.02
3.0	1	0	0.564	0.136	0.436	0.136
4.5	1	0	0.667	0	0.333	0

S4 Table. Time dependent fractional change in area (δ) of the DOPC-GUVs and DOPG/DOPC (40/60)-GUVs induced by 0.5 μg/mL MNPs corresponding to Fig. 9 and 10.

Time (s)	δ for DOPC				δ for DOPG/DOPC (40/60)		
	GUV 1	GUV 2	GUV 3	GUV 4	GUV1	GUV 2	GUV 3
0	0	0	0	0	0	0	0
10	0.17730	-	0.20892	-	-	-	-
13	-	0.19434	-	-	-	-	-
15	-	-	-	0.22923	0.11195	0.07933	0.08513
30	0.15717	0.14851	0.14827	0.15757	0.0594	-0.03090	0.00332
60	0.11121	0.06497	0.06097	0.10599	-0.0064	-0.05202	-0.01130
120	0.06733	0.03650	0.04189	0.05613	-0.02074	-0.05989	-0.00680
180	0.05578	0.02499	0.03621	0.02932	-0.01455	-0.06150	-0.00104
240	0.06254	0.03524	0.04067	0.02427	-0.00924	-0.05822	0.00048
300	0.06254	0.03606	0.05114	0.03120	-0.01037	-0.04934	0.00483
360	0.06947	0.04960	0.05184	0.04165	-0.00391	-0.04936	0.00788
420	0.07924	0.05359	0.06244	0.04576	-0.00007	-0.05143	0.01499
480	0.08757	0.06104	0.06699	0.05029	0.00000	-0.04264	0.01792
540	0.09366	0.06502	0.07338	0.05991	0.00258	-0.03727	0.02075
600	0.09871	0.06918	0.07489	0.06219	0.00636	-0.03542	0.02899

References

1. Zaman MM, Karal MAS, Khan MNI, Tareq ARM, Ahammed S, Akter M, et al. Eco-friendly synthesis of Fe_3O_4 nanoparticles based on natural stabilizers and their antibacterial applications. ChemistrySelect. 2019;4: 7824–7831. doi:10.1002/slct.201901594

2. Karal MAS, Ahammed S, Levadny V, Belaya M, Ahamed MK, Ahmed M, et al. Deformation and poration of giant unilamellar vesicles induced by anionic nanoparticles. Chem Phys Lipids. 2020;230: 104916. doi:10.1016/j.chemphyslip.2020.104916

3. Akter S, Karal MAS, Hasan S, Ahamed MK, Ahmed M, Ahammed S. Effects of cholesterol on the anionic magnetite nanoparticle-induced deformation and poration of giant lipid vesicles. RSC Adv. 2022;12: 28283–28294. doi:10.1039/D2RA03199J

4. Karal MAS, Sultana S, Billah MM, Moniruzzaman M, Wadud MA, Gosh RC. Effects of polyethylene glycol-grafted phospholipid on the anionic magnetite nanoparticles-induced deformation and poration in giant lipid vesicles. PLOS ONE. 2023;18: e0289087. doi:10.1371/journal.pone.0289087

5. Hasan S, Karal MAS, Akter S, Ahmed M, Ahamed MK, Ahammed S. Influence of sugar concentration on the vesicle compactness, deformation and membrane poration induced by anionic nanoparticles. PLOS ONE. 2022;17: e0275478. doi:10.1371/journal.pone.0275478

6. Moniruzzaman M, Karal MAS, Wadud MA, Rashid MMO. Increase in anionic Fe_3O_4 nanoparticle-induced membrane poration and vesicle deformation due to membrane potential – an experimental study. Phys Chem Chem Phys. 2023;25: 23111–23124. doi:10.1039/D3CP02702C

7. Karal MAS, Billah MM, Nasrin T, Moniruzzaman M. Interaction of anionic Fe_3O_4 nanoparticles with lipid vesicles: a review on deformation and poration under various conditions. RSC Adv. 2024;14: 25986–26001. doi:10.1039/D4RA05686H

8. Alfredo Reyes Villegas V, Isaías De León Ramírez J, Hernandez Guevara E, Perez Sicairos S, Angelica Hurtado Ayala L, Landeros Sanchez B. Synthesis and characterization of magnetite nanoparticles for photocatalysis of nitrobenzene. J Saudi Chem Soc. 2020;24: 223–235. doi:10.1016/j.jscs.2019.12.004

9. Razack SA, Suresh A, Sriram S, Ramakrishnan G, Sadanandham S, Veerasamy M, et al. Green synthesis of iron oxide nanoparticles using Hibiscus rosa-sinensis for fortifying wheat biscuits. SN Appl Sci. 2020;2: 898. doi:10.1007/s42452-020-2477-x

10. Yan H, Zhang J, You C, Song Z, Yu B, Shen Y. Influences of different synthesis conditions on properties of Fe3O4 nanoparticles. Mater Chem Phys. 2009;113: 46–52. doi:10.1016/j.matchemphys.2008.06.036

11. Sun J, Zhou S, Hou P, Yang Y, Weng J, Li X, et al. Synthesis and characterization of biocompatible Fe_3O_4 nanoparticles. J Biomed Mater Res Part A. 2007;80A: 333–341. doi:10.1002/jbm.a.30909

12. Iida H, Takayanagi K, Nakanishi T, Osaka T. Synthesis of Fe_3O_4 nanoparticles with various sizes and magnetic properties by controlled hydrolysis. J Coll Interf Sci. 2007;314: 274–280. doi:10.1016/j.jcis.2007.05.047
